# Cardiac MRI predictors of right ventricular dysfunction after the Da Silva cone operation for Ebstein's anomaly

**DOI:** 10.1016/j.ijcchd.2022.100342

**Published:** 2022-01-31

**Authors:** Tarek Alsaied, Carlos Diaz Castrillon, Adam Christopher, Jose Da Silva, Victor O. Morell, Lizabeth Lanford, Bryan H. Goldstein, Brian Feingold, Thomas Seery, Gaurav Arora, Arvind Hoskoppal, Jennifer A. Johnson, Sameh Tadros, Laura J. Olivieri, Luciana De Fonseca Da Silva

**Affiliations:** a Heart Institute, UPMC Children's Hospital of Pittsburgh, Division of Pediatric Cardiology, University of Pittsburgh School of Medicine, Pittsburgh, PA, USA; b DaSilva Center of Ebstein Anomaly, UPMC Children's Hospital of Pittsburgh, University of Pittsburgh School of Medicine, Pittsburgh, PA, USA; c Department of Cardiothoracic Surgery, UPMC Children's Hospital of Pittsburgh, University of Pittsburgh School of Medicine, Pittsburgh, PA, USA; d Department of Radiology, UPMC Children's Hospital of Pittsburgh, University of Pittsburgh School of Medicine, Pittsburgh, PA, USA; e Division of Cardiology, Children's National Hospital, Washington, DC, USA

**Keywords:** Cardiac MRI, Ebstein, Tricuspid valve, Congenital heart disease, Adults with congenital heart disease

## Abstract

**Introduction:**

Despite the clinical benefits of the cone operation for Ebstein's anomaly, significant right ventricular (RV) dysfunction is frequently seen immediately after the procedure and if persistent may portend worse long-term outcomes. In this study we sought to evaluate the predictors of RV dysfunction after the cone operation using preoperative CMR.

**Methods:**

This was a retrospective review of 26 consecutive patients who had the cone operation. Patients with significant RV dysfunction (RVD), defined as moderate or severe dysfunction by discharge echocardiogram, were compared to patients with no or mild dysfunction (no RVD).

**Results:**

The median age at the operation was 12.2 years (interquartile range (IQR): 4.9–31.7 years). Eighteen patients (69%) had RVD. Patients with RVD had worse preoperative RV ejection fraction (36 ​± ​15 vs 49 ​± ​11%, p ​= ​0.02) and a larger cardiothoracic (CT) index (44 ​± ​8 vs 37 ​± ​6, p ​= ​0.03). The tricuspid valve was more severely abnormal in the RVD group with higher rotational angle (45 ​± ​17 vs 23 ​± ​10°, 0.03) and higher displacement index (39 ​± ​18 vs 23 ​± ​12%, p ​= ​0.02). RVD associated with a higher vasoactive inotropic score (P ​< ​0.01) and a trend towards a longer intensive care stay (p ​= ​0.07).

**Conclusion:**

RVD is common after the cone operation and associated with higher need for postoperative inotropes. Predictors include lower preoperative RV ejection fraction, a more dilated heart and more severe tricuspid valve abnormality. Preoperative CMR is an important tool in preoperative assessment and helps predict RVD.

## 1 Introduction

Ebstein's anomaly is the most common congenital malformation of the tricuspid valve with an estimated incidence of 2.4 per 10,000 live births [1]. The etiology is secondary to failure of tricuspid valve delamination from the right ventricle (RV) with underlying RV myopathy. The characteristic features include apical displacement of the functional tricuspid valve orifice, “atrialization” of the RV, fenestrations and tethering of the anterior and inferior leaflets of the tricuspid valve, RV dilation and right atrioventricular junction dilation [1,2].

**Fig. 1:**
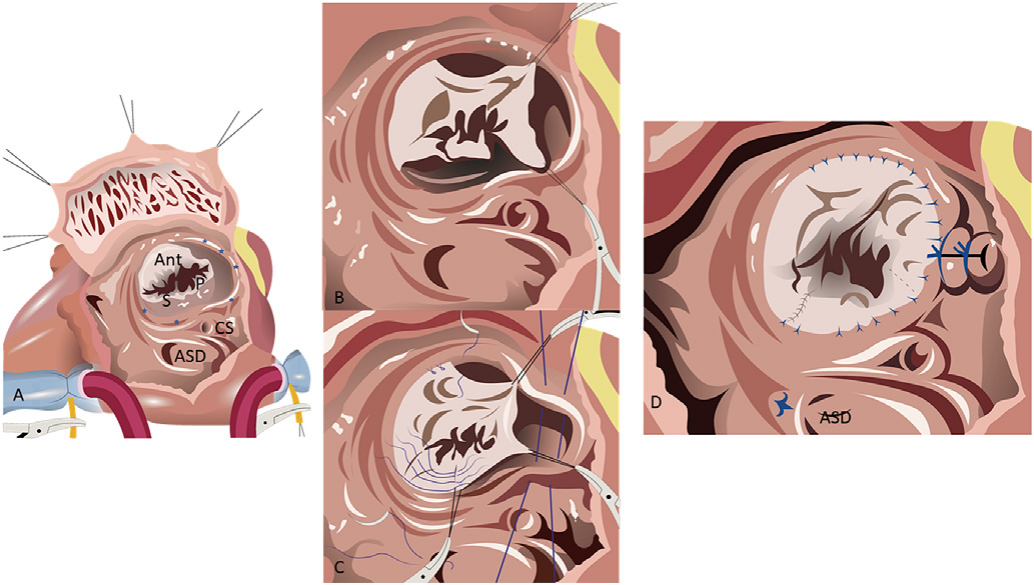
The cone operation of the tricuspid valve. A: The right atrium is open. Cannulas are seen in the caval veins. The anatomical annulus of the tricuspid vale is marked with the blue stars. The tricuspid valve is apically displaced. The leaflets of the tricuspid valve are seen. Ant: represents the anterior leaflet of the tricuspid valve, P is the posterior (inferior) leaflet which is small and apically displaced and S is the septal leaflet of the tricuspid valve. ASD: atrial septal defect and CS is the coronary sinus. B: Detachment of the posterior and anterior leaflets to form a single piece. C: Rotation of the detached valve to the anatomic annulus and plication of the tricuspid valve annulus. D: The complete cone operation with tricuspid valve tissue covering the entire anatomic annulus, plication of the right atrium and closure of the atrial septal defect.

The cone reconstruction technique of the tricuspid valve was developed in 1989 by Dr. Jose Pedro da Silva [3]. The technique involves circumferential leaflet delamination and results in a complete ring of tricuspid valve leaflet tissue around the atrioventricular junction (Fig. 1). This allows for better coaptation and improves regurgitation. The goals of the operation are to obtain a competent tricuspid valve, preserve or improve RV function, and thus decrease the risk of late arrhythmias [4].

**Fig. 2:**
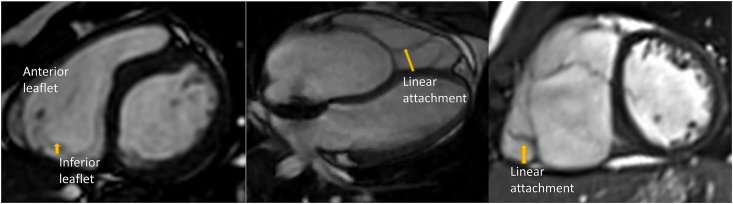
The left panel represents normal focal attachment of the tricuspid valve to the right ventricular free wall in a patient without Ebstein anomaly (arrow). The right two panels represent linear attachment of the tricuspid valve leaflets to the right ventricular free wall.

Cardiac magnetic resonance (CMR) allows for functional and anatomical assessment of the RV and tricuspid valve anomaly and aids surgical planning before the cone operation [5]. A previous study by Johnson et al. showed that preoperative CMR provided additional information in more than three quarters of patients with 69% of the findings altering surgical management [6]. Some anatomical details that can be evaluated by CMR include leaflet attachments of the inferior and anterior leaflets to the RV wall. The type of attachments can be divided into focal attachments and linear attachments. Focal attachments are normal attachments and allows free communications between the atrialized and functional RV. Linear attachments occur when there is complete or partial attachment of the leaflet to a muscular shelf at the connection between the atrialized and functional RV [2] (Fig. 2, Video 1 and 2). Additionally, CMR can measure the degree of displacement and rotation of the tricuspid valve. Finally, CMR gives an accurate assessment of chamber size including atrialized and functional right ventricle and RV function.

Supplementary video related to this article can be found at https://doi.org/10.1016/j.ijcchd.2022.100342

The following are the supplementary data related to this article:

Video 1 1 Video 1

Video 2 2 Video 2

Despite the significant improvement in outcomes and in tricuspid regurgitation after the cone operation, RV dysfunction is frequently seen at discharge echocardiogram. Although RV dysfunction may improve with time, RV dysfunction in the immediate post-operative period may be associated with long term RV dysfunction in some patients. Ventricular dysfunction is a known risk factor for late arrhythmia and sudden cardiac death in patients with Ebstein's anomaly regardless of the surgical intervention status [7]. . Thus, this study aimed to assess the predictors of short-term RV dysfunction in patients after the cone operation using the preoperative CMR. Our hypothesis was that patients with preoperative RV dysfunction by CMR will continue to have RV dysfunction after the cone operation and that patients with more complex valve anatomy (worse displacement, rotation, and linear leaflet attachments) are more likely to have RV dysfunction on post operative echocardiogram.

## 2 Methods

### 2.1 Subjects

A database search identified all patients who had the cone operation at our institution after 2016 who had a CMR study performed before the operation. Subjects were included if ventricular volume and function could be calculated by CMR. The UPMC Children's Hospital of Pittsburgh Institutional Review Board approved this retrospective study and waived the requirement for informed consent.

### 2.2 CMR

CMR studies were performed with 1.5 ​T scanners (Siemens Healthineers, Erlangen, Germany and GE Medical Systems, Milwaukee, Wisconsin) per our institutional protocol [8,9]. If a patient had multiple eligible CMR studies, the most recent CMR study before the cone operation was used for analysis as it is the most pertinent to the current clinical status. Ventricular assessment was performed by a cardiac retro-gated, steady-state free precession cine sequence in ventricular short-axis planes with careful attention to include the entire functional ventricular cavity from base-to-apex. Additionally, a 4-chamber stack was obtained as a second method to evaluate right ventricular size and function.

### 2.3 CMR data analysis

Ventricular volumes of both ventricles were measured by manual tracing of endocardial and epicardial borders at end-diastole (maximal volume) and end-systole (minimal volume) as previously described [[9], [10], [11]]. The results of the right ventricular volumes and function were reported from the 4-chamber stack. All the CMRs were reviewed, and the contours confirmed by a pediatric cardiologist with 4-year CMR experience (TA, ABC). Ventricular volumes were indexed to the body surface area. All analyses were performed using commercially available software (cvi42, Circle Cardiovascular Imaging Inc., Calgary, Canada).

**Fig. 3:**
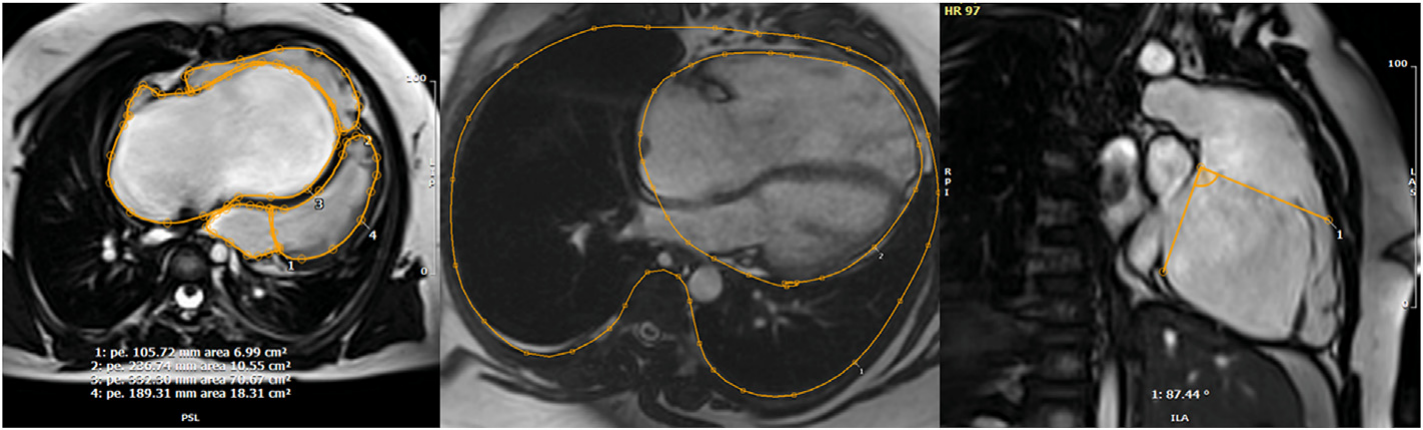
The left image is a 4-chamber view. The Celemajer index ​= ​the area of the right atrium and atrialized right ventricle/(the area of the functional right ventricle ​+ ​left ventricle and left atrium). The middle panel represents the cardiothoracic ratio measurement by dividing the cardiac area over the chest area. The right panel is a right ventricular 3 chamber view to calculate the tricuspid valve rotational angle.

Leaflet attachments of the inferior and anterior leaflets to the right ventricular wall was categorized as either focal or linear attachments. As mentioned above, focal attachments are part of the normal valve apparatus and allow for free communication between the atrialized and functional RV. Linear attachments occur when there is complete or partial attachment of the leaflet to a muscular shelf at the connection between the atrialized and functional ventricle [2] (Fig. 2, Video 1). As most of these patients had severe displacement of the septal leaflet with severe tethering and hypoplasia, the description of this leaflet beyond the displacement index was not possible. To evaluate the reproducibility of the leaflet attachments classification a second cardiologist blindly evaluated a randomly selected 15 CMRs focusing on anterior leaflet attachments. The Celermajer index was calculated as the right atrium plus the atrialized portion of the RV/(functional RV ​+ ​left atrium ​+ ​left ventricle) from the 4-chamber view in diastole [12] (Fig. 3). The RV outflow tract rotational angle was calculated from the RV 3-chamber view as previously described [12,13] (Fig. 3). Briefly, the end-systolic frame was selected from the cine loop, and the rotational angle was measured with the fulcrum of the angle positioned at the basal superior “hinge-point” of the valve in this cine view, anterior to the aortic valve. The cardiothoracic (CT) index was calculated from the 4-chamber stack view in diastole at the slice with largest cardiac area (Fig. 3). The CT index was calculated as 100∗ cardiac area/thoracic area. The displacement index was calculated as the 100∗ the maximal displacement of the septal leaflet of the tricuspid valve/base to apex diameter of the right ventricle measured from a 4-chamber view in diastole. The base level was determined as the level of the right atrioventricular groove.

### 2.4 Clinical data

Clinical and demographic data included age at surgery and previous procedures. The discharge echocardiogram was evaluated for RV dysfunction, tricuspid valve disease severity and left ventricular dysfunction. RV dysfunction was evaluated by conventional echocardiographic criteria using the fractional area change, tricuspid annular plane systolic excursion, and qualitative assessment. Significant RV dysfunction (RVD) was considered as moderate or more ventricular dysfunction on discharge echocardiogram. The RVD group was compared to the group with no or mild RV dysfunction on discharge echocardiogram (no RVD) regarding clinical and preoperative imaging findings. Additionally, to evaluate the clinical impact of RVD we compared the hospital and cardiac intensive care unit (CICU) length of stay (LOS). The peak dose of the inotropes and vasoactive medications and the duration of inotropic support were collected. The two inotropes used post operatively in this cohort were milrinone and epinephrine. No other inotropes or vasoactive medications were used in our cohort. The vasoactive inotropic score (VIS) was also calculated as previously described and was compared between patients with and without RVD [14]. Briefly, as only milrinone and epinephrine were used post-operatively the VIS score was calculated as [10 × milrinone dose in (mcg/kg/min)] ​+ ​[100 × epinephrine dose (mcg/kg/min)] [14].

### 2.5 Statistical analysis

Categorical variables were summarized using frequencies and percentages and compared for subjects in the two groups (RVD and no RVD) using a Fisher exact test. Continuous variables were summarized using either the mean and standard deviation or median and interquartile range (IQR), as appropriate, and were compared for subjects in the two groups using the unpaired *t*-test or Wilcoxon rank-sum test.

Additionally, if continuous variables were associated with RVD, cut points were obtained using Youden's index that allowed maximal discrimination by the receiver operator characteristic (ROC) curve analyses to improve interpretation of the data and determine the ability of CMR imaging indices to predict subjects with RVD. The area under the curve (AUC) from ROC analysis was reported to evaluate the ability of a certain preoperative CMR parameter to predict post-operative RVD. Due to the wide age range, a logistic regression was used to determine the odds ratios (OR) for different preoperative CMR predictors of RVD after adjusting for age at the operation. Finally, to evaluate the agreement of the two readers regarding leaflet attachments, Fleiss inter-rater kappa test was used.

A two-sided p-value of less than 0.05 was considered statistically significant. Statistical analyses were performed using IBM SPSS Statistics for Windows (version 28.0, Armonk, NY) and JMP® (version 15, SAS Institute Inc., Cary, NC).

## 3 Results

### 3.1 Population characteristics

A total of 26 patients were included in the study. The cohort consisted of 16 females and 10 males. The median age at the cone operation was 12.2 years (IQR: 4.9–31.7 years). Seven patients (27%) had previous operations. These included previous tricuspid valve intervention in 2 patients, Glenn operation in 3 patients, aortopulmonary shunt in 4 patients and Starnes operation in 1 patient. These procedures are not mutually exclusive. There was no in-hospital mortality. One patient who is a 35-year-old with history of ventricular and supraventricular tachycardia and multiple ablations required pacemaker implantation after he had a cone operation and a maze procedure. One patient required reoperation for valve repair one week after the original operation.

### 3.2 Findings on discharge echocardiogram

The median time between the cone operation to discharge echocardiogram was 4 days (IQR: 2.5–7.5 days). Eighteen patients (69%) had RVD by discharge echocardiogram while 8 patients (31%) had no RVD (normal or only mild RV dysfunction). In the RVD group, the RV function was moderately depressed in 12 (46%) and severely depressed in 6 patients (23%). There was no difference in the age of operation between the patients with RVD and with no RVD (16.1 ​± ​16.7 vs 24.3 ​± ​16.9 years, p ​= ​0.28).

The severity of tricuspid regurgitation was trivial in 6 (24%), mild in 14 (52%) and moderate in 6 (24%). Sixteen (61%) patients had no, or trivial tricuspid stenosis and 10 patients (39%) had mild tricuspid stenosis. The left ventricular function was normal in 24 patients and mildly depressed in 2 patients. One patient had a small pericardial effusion.

### 3.3 Preoperative CMR findings in the two groups

Patients with RVD had worse RV ejection fraction on preoperative CMR (36 ​± ​15 vs 49 ​± ​11%, p ​= ​0.02) (Table 1). Additionally, they had more severely dilated hearts with larger CT indices (44 ​± ​8 vs 37 ​± ​6, p ​= ​0.03) and Celermaier indices (0.97 ​± ​0.74 vs 0.46 ​± ​0.33, p ​= ​0.05). The indexed left ventricular (LV) end-diastolic volume was smaller in the RVD group (50 (45–66) vs 70 (57–81) ml/m2, p ​= ​0.02). There was a trend towards a higher indexed functional RV end-diastolic volume (181 ​± ​116 vs. 121 ​± ​56 ml/m2, p ​= ​0.09) in patients with RVD.

**Table 1:** Differences between patients with and without significant right ventricular dysfunction.

	Right ventricular dysfunction			P value
	Yes (n ​= ​18)		No (n ​= ​8)	
Age at repair	16.1 ​± ​16.7	24.3 ​± ​16.9		0.28
Cardiothoracic index by CXR (%)	69 ​± ​13	56 ​± ​12		0.02
**Preoperative CMR**				
Preoperative RV ejection fraction (%)	36 ​± ​15	49 ​± ​11		0.02
RV indexed end diastolic volume (ml/m2)	181 ​± ​116	121 ​± ​56		0.09
RV indexed end systolic volume (ml/m2)	123 ​± ​91	59 ​± ​26		0.02
LV indexed end diastolic volume (ml/m2)	50 (45–66)	70 (57–81)		0.02
LV indexed end systolic volume (ml/m2)	26 ​± ​13	42 ​± ​36		0.26
Preoperative LV ejection fraction (%)	57 ​± ​12	59 ​± ​8		0.60
Linear attachments of the anterior leaflet (%)	78%	25%		0.20
Linear attachments of the inferior leaflet (%)	92%	20%		0.03
Celemajer index	0.97 ​± ​0.74	0.46 ​± ​0.33		0.05
Cardiothoracic index	44 ​± ​8	37 ​± ​6		0.03
Tricuspid valve rotational angle	45 ​± ​17	23 ​± ​10		0.03
Tricuspid regurgitant fraction (%)	44 ​± ​19	48 ​± ​21		0.77
Displacement index (%)	39 ​± ​18	23 ​± ​12		0.02
**Follow up echocardiogram**				
Tricuspid regurgitation severity moderate or more (%)	17%	38%		0.12
Tricuspid stenosis moderate or more (%)	0%	0%		1.0

The tricuspid valve was also anatomically more abnormal in the RVD group with higher displacement index (39 ​± ​18 vs 23 ​± ​12, p ​= ​0.02) and higher rotational angle (45 ​± ​17 vs 23 ​± ​10°, p ​= ​0.03).

Patients with RVD by discharge echocardiogram were more likely to have linear attachments of the inferior leaflet (92% vs 20%, p ​= ​0.03). Anterior leaflet linear attachment was more common in the RVD group but was not statistically significant (78% vs 25%, p ​= ​0.20). There was good agreement between both readers regarding leaflet attachment (kappa ​= ​0.7, p ​= ​0.007). There was no difference in tricuspid regurgitant fraction by preoperative CMR between the two groups (44 ​± ​19 vs 48 ​± ​21%, p ​= ​0.77).

### 3.4 Receiver operator characteristics analysis

AUCs for different significant continuous variables to predict post-operative RVD are summarized in Table 2. CT index and rotational angle had the largest AUC of 0.88 each, followed by displacement index and LV end-diastolic volume 0.80 each. RV ejection fraction has an AUC of 0.76 and Celermajer index has an AUC of 0.74. The sensitivity, specificity positive and negative predictive values of different thresholds obtained from the analysis are summarized in Table 2. Preoperative moderate or severe RV dysfunction by CMR (ejection fraction <35%) has a positive predictive value of 100% for post operative RVD.

**Table 2:** Area under the curve, sensitivity and specificity of thresholds obtained from the receiver operator characteristics curve analysis.

Parameter	Area under the curve	Threshold	Sensitivity (%)	Specificity (%)	Positive predictive value (%)	Negative predictive value (%)
**Preoperative RV ejection fraction (%)**	0.76	35	50	100	100	47
**RV indexed end systolic volume (ml/m2)**	0.75	91	53	100	100	49
**LV indexed end diastolic volume (ml/m2)**	0.80	53	63	100	100	55
**Celemajer index**	0.74	0.58	75	75	87	57
**Cardiothoracic index by CMR (%)**	0.88	44	63	100	100	55
**Tricuspid valve rotational angle (degree)**	0.88	28	100	75	87	100
**Displacement index (%)**	0.80	23	92	66	81	79

After adjusting for age (Table 3), a higher preoperative RV ejection fraction was associated with decreased odds for RVD (odds ratio [OR]: 0.61 per 5% increase in ejection fraction, confidence interval [CI]: 0.48–0.76, p ​= ​0.04). In contrast the odds for RVD were higher with increased CT index (OR: 1.37 per 1% increase, CI: 1.05–1.75, p ​= ​0.02), increased displacement index (OR: 2.44 per 10% increase, CI: 1.01–6.23, p ​= ​0.02), increased rotational angle (OR: 3.23 per 5% increase, CI: 1.01–17.2, p ​= ​0.02) and with linear attachment of the inferior leaflet (OR: 10.9, CI: 1.01–131, p ​= ​0.03).

**Table 3:** Logistic regression for imaging parameters. All odds ratios are adjusted for age.

Parameter	Odds ratios	Confidence Interval	P value
**Pre-operative RV ejection fraction (per 5% increase)**	0.61	0.48–0.76	0.04
**Cardiothoracic index (per 1% increase)**	1.37	1.05–1.75	0.02
**Displacement index (per 10% increase)**	2.44	1.01–6.23	0.02
**Rotational angle (per 5° increase)**	3.23	1.01–17.2	0.02
**Linear attachment of the inferior leaflet**	10.9	1.01–131	0.03

### 3.5 RVD and clinical outcomes

Patients with RVD by discharge echocardiogram received a higher peak dose of milrinone (0.52 ​± ​0.31 vs 0.20 ​± ​0.15 mcg/kg/min, p ​< ​0.01) and a longer duration of milrinone infusion (78 ​± ​71 vs 14 ​± ​8 ​h, p ​< ​0.01). VIS was also higher in the RVD group (8.7 ​± ​4.1 vs 4.3 ​± ​2.5, p ​< ​0.01). The CICU LOS was longer in the RVD group although was not statistically significant [3.0 (IQR: 2.0–6.5) vs 1.5 (IQR: 1.0–5.0) day, p ​= ​0.07]. There was no statistically significant difference in hospital LOS between the RVD group and the group with no RVD (6 (IQR: 4.8–9.2) vs 4 (IQR:2.3–10.3) day, p ​= ​0.23).

## 4 Discussion

**Fig. 4:**
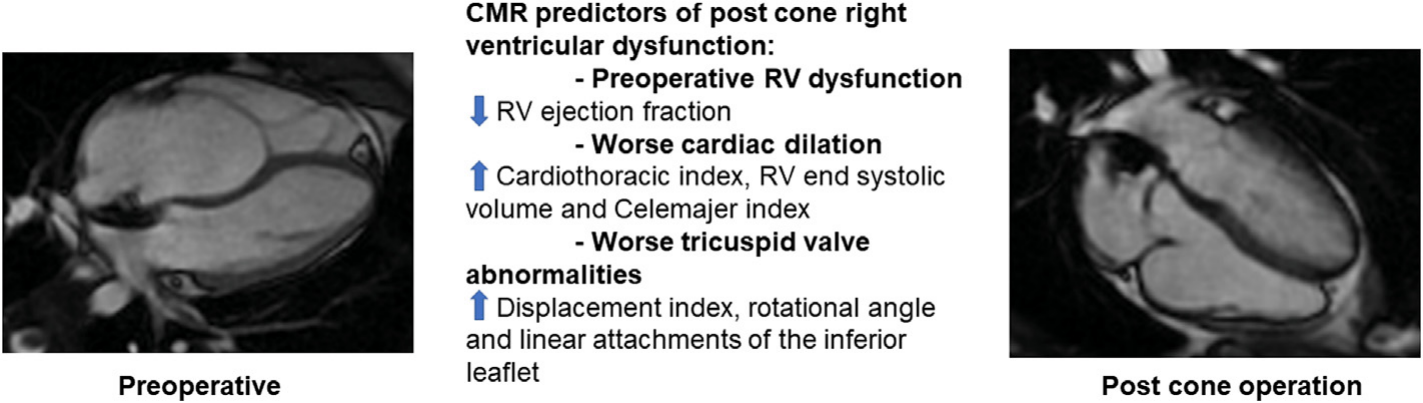
A graphical summary of the main study findings. RV: right ventricle.

This study compared the preoperative CMR characteristics of patients after the cone operation with RVD on discharge echocardiogram and patients with no RVD (Fig. 4, Video 2). The study found that patients with RVD had 1) more complex tricuspid valve anatomy (higher displacement index, rotational angle, and linear attachments of the inferior leaflet), 2) lower preoperative RV ejection fraction, 3) larger hearts with higher CT index and Celemajer index and 4) lower left ventricular end diastolic volume. RVD was associated with increased need for inotropes after surgery and a trend towards longer CICU LOS although not with the overall hospital LOS.

Post-operative RVD is common after the cone operation despite the significant clinical improvement after surgery [15]. RVD is multifactorial, but most likely is related to incorporation of the atrialized RV into the functional RV chamber during the procedure. The pre-operative functional RV is ill-prepared due to chronic volume overload. This is exacerbated by the stresses of cardiopulmonary bypass, electrical conduction abnormalities, and possible peri-operative ischemia [16]. Early RVD may improve with time but, RVD remains common years after the cone operation [15,17]. Recent studies have shown progressive improvement in biventricular function after the cone operation, although RVD is an important risk factor for sudden death after the surgical repair of the tricuspid valve [15,18]. Post operative RVD is associated with increased need for inotropic support and a trend to a longer CICU stay in our study. Thus, it is important to recognize the predictors of significant RVD.

This study found that more abnormal tricuspid valves with worse displacement, rotation and linear attachments is associated with increased risk of RVD. This makes intuitive sense as more abnormal valves will have larger atrialized RV and require longer and more complex surgery [20,21]. Additionally separating the linear attachments of the valve leaflets may result in further thinning of the functional RV or interfere with the stability of the free wall and unmask RV functional abnormalities [22].

Additionally, the study shows that a dysfunctional RV and dilated heart are risk factors for early post-operative significant RVD. Previous studies showed that progressive RV dilation and dysfunction is common in Ebstein's anomaly [19]. As symptoms of RV failure are rare and are typically late findings, referral for surgical repair may be delayed which may result in significant post-operative RVD due to the chronic RV remodeling [19].

One important implication of this study is the importance of preoperative CMR before the cone operation [17]. Findings of CMR help predict patients who will develop ventricular dysfunction and provided important anatomical and functional data. It is well known that CMR is the imaging modality of choice to evaluate RV size and function given the variable geometry of the right ventricle and sub-sternal location which complicates evaluation by 2-dimensional echocardiography [23]. More recently CMR has played an important complimentary role to echocardiography assessing the tricuspid valve [24]. Our findings suggest that CMR provides important additional data related to tricuspid valve anatomy and the degree of cardiac dilation and ventricular function. This supports the importance of preoperative CMR to guide an individualized surgical plan for each patient. Additionally, our findings are encouraging showing that RVD post operatively did not result in increased hospital LOS. This is likely because that despite the lower ejection fraction after the cone operation the cardiac output increases in these patients due to the improved diastolic ventricular interaction and the relief of tricuspid regurgitation. Additionally, the pulmonary vascular resistance is likely normal in most of our patients and thus RVD did not translate into low cardiac output. This suggests that the RVD seen post cone repair is well tolerated and at least on the short term did not worsen the prognosis. Long term studies are needed to understand the natural history of RVD and its association with long-term outcomes.

## 5 Limitations

As a single center cohort, further studies are needed to validate the results in an independent cohort of patients. By design, the cohort included only subjects who have undergone CMR before the cone operation, which may lead to potential selection bias, though pre-operative CMR has become our institutional standard regardless of anatomy. Finally, we did not evaluate the long-term outcomes for RVD, and this will be the focus of our future research.

In conclusion, in this modern cohort of patients with CMR before the cone operation, we demonstrate that RVD is common immediately after the cone operation. Predictors of significant RVD include lower preoperative RV ejection fraction, a more dilated heart and more severe tricuspid valve abnormality. Preoperative CMR is an important tool in the assessment and may help predict patients who develop post-operative ventricular dysfunction.

## Funding

No authors report any funding related to this research.

## Conflicts of interest

None.

## References

[1] Paranon S., Acar P. (2008). Ebstein's anomaly of the tricuspid valve: from fetus to adult: congenital heart disease. Heart.

[2] Leung M.P., Baker E.J., Anderson R.H., Zuberbuhler J.R. (1988). Cineangiographic spectrum of Ebstein's malformation: its relevance to clinical presentation and outcome. J Am Coll Cardiol.

[3] Liu J., Qiu L., Zhu Z., Chen H., Hong H. (2011). Cone reconstruction of the tricuspid valve in Ebstein anomaly with or without one and a half ventricle repair. J Thorac Cardiovasc Surg.

[4] Dearani J.A. (2020). Ebstein repair: how I do it. JTCVS Tech.

[5] Beroukhim R.S., Jing L., Harrild D.M. (2018). Impact of the cone operation on left ventricular size, function, and dyssynchrony in Ebstein anomaly: a cardiovascular magnetic resonance study. J Cardiovasc Magn Reson.

[6] Johnson J.T., Molina K.M., McFadden M., Minich L.L., Menon S.C. (2014). Yield of cardiac magnetic resonance imaging as an adjunct to echocardiography in young infants with congenital heart disease. Pediatr Cardiol.

[7] Waldmann V., Khairy P. (2018). Ventricular arrhythmias and sudden death in patients with Ebstein anomaly: insights from a retrospective cohort study. J Thorac Dis.

[8] Geva T., Sandweiss B.M., Gauvreau K., Lock J.E., Powell A.J. (2004). Factors associated with impaired clinical status in long-term survivors of tetralogy of Fallot repair evaluated by magnetic resonance imaging. J Am Coll Cardiol.

[9] Geva T. (2011). Repaired tetralogy of Fallot: the roles of cardiovascular magnetic resonance in evaluating pathophysiology and for pulmonary valve replacement decision support. J Cardiovasc Magn Reson : Off J Soc Cardiovasc Magnet Res.

[10] Valente A.M., Gauvreau K., Assenza G.E. (2014). Contemporary predictors of death and sustained ventricular tachycardia in patients with repaired tetralogy of Fallot enrolled in the INDICATOR cohort. Heart (British Cardiac Soc).

[11] Fratz S., Chung T., Greil G.F. (2013). Guidelines and protocols for cardiovascular magnetic resonance in children and adults with congenital heart disease: SCMR expert consensus group on congenital heart disease. J Cardiovasc Magn Reson : Off J Soc Cardiovasc Magnet Res.

[12] Hughes M.L., Bonello B., Choudhary P., Marek J., Tsang V. (2019). A simple measure of the extent of Ebstein valve rotation with cardiovascular magnetic resonance gives a practical guide to feasibility of surgical cone reconstruction. J Cardiovasc Magn Reson.

[13] Celermajer D.S., Cullen S., Sullivan I.D., Spiegelhalter D.J., Wyse R.K., Deanfield J.E. (1992). Outcome in neonates with Ebstein's anomaly. J Am Coll Cardiol.

[14] Gaies M.G., Jeffries H.E., Niebler R.A. (2014). Vasoactive-inotropic score is associated with outcome after infant cardiac surgery: an analysis from the pediatric cardiac critical care consortium and virtual PICU system registries. Pediatr Crit Care Med.

[15] Lianza A.C., Rodrigues A.C.T., Mercer-Rosa L. (2020). Right ventricular systolic function after the cone procedure for Ebstein's anomaly: comparison between echocardiography and cardiac magnetic resonance. Pediatr Cardiol.

[16] Perdreau E., Tsang V., Hughes M.L. (2018). Change in biventricular function after cone reconstruction of Ebstein's anomaly: an echocardiographic study. Eur Heart J Cardiovasc Imag.

[17] Lange R., Burri M., Eschenbach L.K. (2015). Da Silva's cone repair for Ebstein's anomaly: effect on right ventricular size and function. Eur J Cardio Thorac Surg.

[18] Neijenhuis R.M.L., Tsang V.T., Marek J. (2021). Cone reconstruction for Ebstein anomaly: late biventricular function and possible remodeling. J Thorac Cardiovasc Surg.

[19] Raju V., Dearani J.A., Burkhart H.M. (2014). Right ventricular unloading for heart failure related to Ebstein malformation. Ann Thorac Surg.

[20] Stephens E.H., Dearani J.A., Qureshi M.Y., Ammash N., Maleszewski J.J. (2020). The congenital tricuspid valve spectrum: from Ebstein to dysplasia. World J Pediatr Congen Heart Surg.

[21] Holst K.A., Dearani J.A., Said S. (2018). Improving results of surgery for Ebstein anomaly: where are we after 235 cone repairs?. Ann Thorac Surg.

[22] da Silva J.P., Baumgratz J.F., da Fonseca L. (2007). The cone reconstruction of the tricuspid valve in Ebstein's anomaly. The operation: early and midterm results. J Thorac Cardiovasc Surg.

[23] Alsaied T., Geva T., Graf J.A., Sleeper L.A., Marie Valente A. (2021). Biventricular global function index is associated with adverse outcomes in repaired tetralogy of fallot. Circ Cardiovasc Imag.

[24] Wang T.K.M., Akyuz K., Reyaldeen R. (2021). Prognostic value of complementary echocardiography and magnetic resonance imaging quantitative evaluation for isolated tricuspid regurgitation. Circ Cardiovasc Imag.

